# Association study of candidate genes for susceptibility to Kashin-Beck disease in a Tibetan population

**DOI:** 10.1186/s12881-017-0423-6

**Published:** 2017-06-26

**Authors:** Zhengfu Tai, Lulin Huang, Fang Lu, Yi Shi, Shi Ma, Jing Cheng, He Lin, Xin Liu, Yuanfeng Li, Zhenglin Yang

**Affiliations:** 1 The Key Laboratory for Human Disease Gene Study, Hospital of University of Electronic Science and Technology of China and Sichuan Provincial People’s Hospital, Chengdu, Sichuan China; 2 Chengdu Institute of Biology, Chinese Academy of Sciences, Chengdu, Sichuan China; 3 School of Medicine, University of Electronic Science and Technology of China, Chengdu, Sichuan China; 4 Sichuan Translational Medicine Research Hospital, Chinese Academy of Sciences, Chengdu, Sichuan China; 5 Center for Human Molecular Biology & Genetics, Hospital of University of Electronic Science and Technology of China and Sichuan Provincial, People’s Hospital, 32 The First Ring Road West 2, Chengdu, Sichuan 610072 China

**Keywords:** Kashin-Beck Disease, Osteoarthritis, *FRZB*, *ASPN*, *COL10A1*, *HABP2*

## Abstract

**Background:**

Many osteoarthritis (OA) susceptibility genes have been identified in recent years. Given the overlap in the phenotype of joint inflammation between OA and Kashin-Beck disease (KBD), the aim of this study is to explore whether the reported OA susceptibility genes and two genes that may link to OA pathophysiology are associated with KBD in the Tibetan population.

**Method:**

Fifteen single-nucleotide polymorphisms (SNPs) in 12 candidate genes previously reported as OA susceptibility loci were selected for investigation. Genotyping was performed using the SNaPshot method for these SNPs in a Tibetan population composed of 849 KBD patients and 565 normal controls. Meanwhile, the coding regions of two genes, *COL10A1* and *HABP2*, which may involve in the pathological mechanism of OA/KBD, were sequenced by Sanger sequencing to identify susceptibility coding variants for KBD in the Tibetan population.

**Results:**

The two arthritis-susceptible candidate SNPs, rs7775 (p.Arg324Gly) in the *FRZB* gene and rs7033979 in the *ASPN* gene, showed associations with KBD (OR = 1.568, *P* = 4 × 10^−3^ and OR = 0.744, *P* = 8 × 10^−3^, respectively). The coding variants rs142463796 (p.Asp128Asn) and rs2228547 (p.Gly545Arg) in the *COL10A1* gene (OR = 9.832 and *P* = 6 × 10^−3^ and OR = 1.242, *P* = 0.043, respectively) and rs548354451 (p.Asp272Glu) in the *HABP2* gene (OR = 2.813, *P* = 0.010) were associated with KBD patients.

**Conclusion:**

These finding suggested that rs7775 in the *FRZB* gene may increase susceptibility to KBD, while rs7033979 in the *ASPN* gene may play a protective role in susceptibility to KBD in Tibetans. Moreover, genetic variants in chondrogenesis-related genes *COL10A1* and *HABP2* may play a role in the risk of developing KBD in the Tibetan population.

**Electronic supplementary material:**

The online version of this article (doi:10.1186/s12881-017-0423-6) contains supplementary material, which is available to authorized users.

## Background

Kashin-Beck disease (KBD) was first hinted as an endemic blight in 1849 by a Russian surveyor who noted that people in villages along the Urov River suffered bone deformities. A few years later, a Cossack doctor, Nikolai Kashin, described Urov disease [1]. In 1906, another Cossack doctor, Evgeny Beck, documented cases in the monograph *Osteoarthritis Deformans Endemica*. After that, this type of joint deformity was called “Kashin-Beck Disease” [2]. More than 30 million people live in the endemic areas (northeastern to southwestern China, extending to southeastern Siberia and North Korea) [1]. China is the country with the largest incidence of KBD in the world, and the Tibetans are the people most affected by KBD in China. The prevalence of KBD in Tibetans peaked in the late 1950s, when in many severely hit villages, 60–90% of children showed signs of KBD [3, 4]. The incidence has declined steadily since 2000 due to a massive effort launched to stamp out KBD, including relocating populations to a non-endemic region, but in 2013, there remained 0.64 million patients with KBD and 1.16 million at risk in 377 counties of 13 provinces or autonomous regions in China [5].

KBD is a chronic osteoarthropathy combined with disturbances of flexion and extension in the ankles, knees, wrists and elbows, enlarged inter-phalangeal joints et al. Severely affected cases are characterized by disproportionate, stunted growth with associated joint deformity. The basic pathologic feature of KBD is the death of cartilage cells in the growth plate and articular surface, but its etiology has not been fully defined yet. The general presumption is that risk factors for this disease are nutritional deficiency of selenium and iodine and environmental contamination with mycotoxins and fulvic acid (FA). Selenium and/or iodine have been considered the most important deficiencies associated with KBD [6, 7]. Mycotoxins produced by fungi can contaminate grain, which may cause KBD, because mycotoxins cause lesions in cartilage tissues, especially in physeal cartilage [8]. FA as an exogenous free radical carrier, may accumulate on cartilage cells and lead to severe cartilage damage. Thus, high concentrations of FA in drinking water also have been implicated in the disease [9]. Cold and hypoxia also may be precipitating factors for KBD, consistent with the fact that all KBD-endemic regions are cold and/or hypoxic.

An increasing body of evidence suggests that environmental factors alone cannot account for the etiology of KBD [10]. In the hunt for genetic suspects, several studies have shown that genetic factors play an important role in KBD pathogenesis. In 2010, Xiong et al. showed that the polymorphism (rs1050450) of selenoprotein gene *GPX1* was significantly associated with KBD in a Han Chinese population [11]. However, in our previous study of the Tibetan population, the single single-nucleotide polymorphism (SNP) (rs1050450) of the *GPX1* gene was not significantly associated with KBD. Furthermore, haplotype analysis of SNPs rs1050450, rs1800668 and rs3811699 in the *GPX1* gene showed significant association with KBD [7]. These results suggested that the *GPX1* gene might play a protective role in susceptibility to KBD. In 2011, we reported that four SNPs (rs6457617, rs6457620, rs9275295 and rs7745040) in *HLA-DRB1* gene locus were associated with KBD in the Tibetan population [12]. This result was confirmed in a different population by an independent group, revealing that two polymorphisms (rs7745040 and rs9275295) in the *HLA-DRB1* gene and one polymorphism (rs9473132) in *CD2AP* gene have a significant statistical association with KBD [13]. Most recently, *ITPR2* and *COL9A1* were identified as susceptibility genes for KBD in Han Chinese [14, 15].

These studies suggested that genetic variants played an important role in KBD. Given that KBD has been found to have overlapping phenotypes and pathologic changes with osteoarthritis (OA), we suspected that OA-related gene variants might play an important role in the etiology and pathogenesis of KBD, so we analyzed the association of 15 SNPs in 12 OA-related genes with KBD in a Tibetan population.

In addition, the genes that code for structural proteins of the extracellular matrix (ECM) of the cartilage may play an important role in the development of KBD, especially the *COL10A1* gene, which codes for collagen type X. *COL10A1* has been proved to be a direct transcriptional target of *RUNX2* during chondrogenesis. Immunohistological studies localized type X collagen exclusively in the zone of hypertrophic and calcifying cartilage [16]. And it was reported that *Col10a1-Runx2* transgenic mice with delayed chondrocyte maturation were less susceptible to developing osteoarthritis [17]. Possible involvement of the *HABP2* gene in KBD was suggested based on the following concepts. The *HABP2* gene encodes an extracellular serine protease that binds to hyaluronic acid (also known as hyaluronan, HA). Both HA and HA binding protein (HABP) are important components of articular cartilage whose damage is the principal pathologic feature of KBD [18]. Intra-articular hyaluronic acid (IAHA) injection has been used to treat patients with KBD [19]. Therefore, we also investigated the potential association between KBD susceptibility and variants in *COL10A1* and *HABP2* genes by directly sequencing the exons of both genes.

## Methods

### Subjects and clinical examination

Approval for the study was provided by the Institutional Review Boards of Sichuan Provincial People’s Hospital. KBD patients and controls in this study were recruited from the Tibetan population in the same endemic villages in SongPan, RuoErGai and HongYuan counties in the Aba Tibetan Autonomous prefecture of Sichuan Province, China. All subjects signed informed consent forms prior to participation in the study.

KBD was diagnosed by physical examination and X-ray radiographs when a subject was more than 5 years old, had persistent pain, limitation of mobility and deformity of the shoulders, wrists, fingers, toes, ankles and knees [20], but did not have other types of arthritis, such as rheumatoid arthritis (RA) and OA, or local inflammation and a history of trauma [1, 6, 21]. The diagnosis of KBD was made in accordance with the KBD Chinese diagnosis criteria (GB16003-1995). Anteroposterior radiograph of the right hand of each patient was first taken with portable x-ray equipment. If the patient was classified as the first, second or third stage of KBD the anteroposterior and the lateral radiograph of the elbow, knee, ankle, hip were added. The subject had to have at least one of the following radiological signs was included: irregular erosions of the carpal/tarsal bones or the metacarpal/metatarsal bones and phalanges, irregular metaphyseal widening, cone-shaped epiphyses of phalanges [6]. Controls were individuals from the same regions with normal joint examinations and no other bone or joint diseases.

### Selection of candidate genes and single-nucleotide polymorphism (SNP)

Selection of candidate genes was orientated toward the search for alterations in a gene that might be related to KBD and chondrodysplasia based on its function and possible pathological role. SNPs were selected for genotyping if they were considered to have confirmed associations or demonstrated suggestive evidence of association with OA in previous studies.

### Genotyping

Venous blood was obtained from each subject and collected in an EDTA tube. Genomic DNA was purified using a Gentra Puregene Blood DNA kit (Minneapolis, MN). Genotyping was performed by the dye terminator-based SNaPshot method (Applied Biosystems, Foster City, CA). Additional file 1: Table S1 lists PCR and SNaPshot-specific primers. SNP analysis was performed on the ABI 3730 DNA analyzer (Applied Biosystems). Genotypes of the SNPs were determined by Gene mapper software (Applied Biosystems).

### Exon sequencing

Primers were designed using Exon Primer (http://ihg.gsf.de/ihg/ExonPrimer.html) to amplify the exons of genes and at least 50 bp flanking the boundary of intron-exon junctions of each exon (Additional file 2: Table S2). Amplified exons then were sequenced using the ABI Big Dye on the ABI3730 DNA analyzer.

### Statistical analysis

The Hardy-Weinberg equilibrium (HWE) of each SNP was tested by the goodness-of-fit *χ*2 test to compare the expected frequencies of genotypes in controls, and SNPs with *P*-values > 0.01 were considered to be within the HWE [22]. *P* values of the SNPs were calculated using an additive model. Odds ratios of the alleles and genotypes were estimated by the *χ*2 test. *P*-values < 0.05 were considered statistically significant. All statistical analyses were performed using SPSS version 17.0 software.

## Results

### Clinical features of the study subjects

**Fig. 1:**
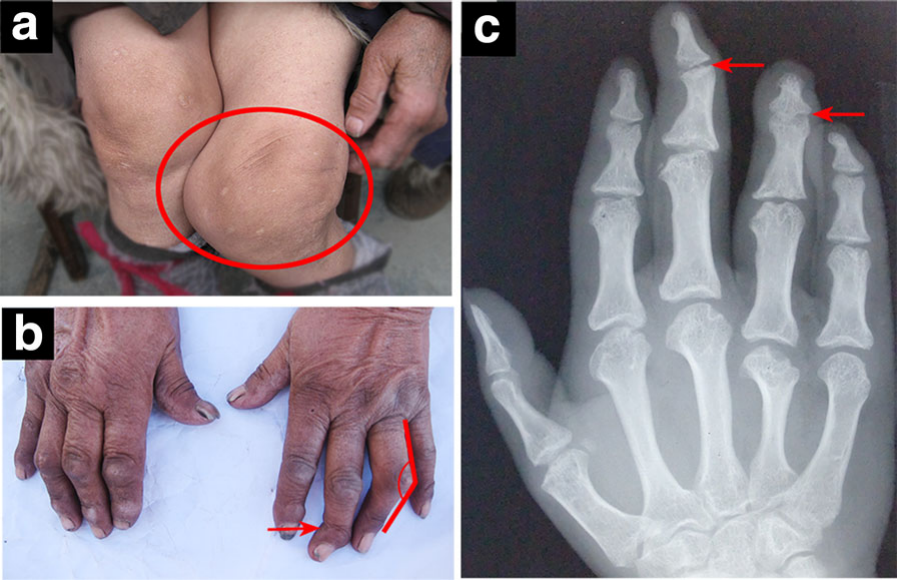
Clinical features of patients with Kashin-Beck disease (KBD). **a** and **b** A patient with KBD shows obvious flexion and extension in the knees (*circled area*) and inter-phalangeal joints (*arrow and angle labeled area*), with deformity and limited mobility. **c** The x-ray radiographs of a KBD patient with irregular, enlarged hand inter-phalangeal joints

The study included 849 KBD patients and 565 controls. Table 1 lists information about patients and controls. There was no significant difference in gender distribution between KBD patients and controls (*χ*2 = 3.51, *P* > 0.05). KBD patients ranged from 5 to 75 years old, and the mean age was 53.7 ± 9.8 years for males and 53.5 ± 10.5 years for females. Controls ranged from 40 to 72 years old, with mean ages of 54.6 ± 10.0 years for males and 54.0 ± 9.5 years for females. As shown in Fig. 1a–c), the main features of KBD patients include flexion and extension in the knees, ankles, wrists and elbows and/or enlarged inter-phalangeal joints and so on, with the possibility of OA and/or RA being ruled out. No signs of KBD, OA or other autoimmune disease were detected in the controls.

**Table 1:** Characteristics of Kashin-Beck disease patients and controls

Samples	No. of Samples			Age, Mean ± SD M/F (years)
	Total	Male	Female	
Case	849	489	360	53.7 ± 9.8/53.5 ± 10.5
Control	565	296	269	54.6 ± 10.0/54.0 ± 9.5

### Association of SNPs in *FRZB* and *ASPN* genes with KBD

This study selected 15 SNPs (mapping to 12 genes) reported previously as suggestive or confirmed susceptibility loci for OA (Table 2) to investigate whether they were susceptibility loci for KBD in the Tibetan population. All 15 SNPs had a genotyping success rate > 96% and accuracy as judged by random re-genotyping of 10% of the samples by direct sequencing analysis. Table 3 presents the case–control comparison of allele frequencies for the 15 SNPs. Significant differences in the minor allele frequencies between the KBD cases and controls were detected at rs7775 of the *FRZB* gene and rs7033979 of the *ASPN* gene. Results show that the frequency of the minor allele “G” of rs7775 (p.Arg324Gly) in the *FRZB* gene was higher in patients with KBD than in controls (OR = 1.568, 95% CI = 1.151–2.136, *P* = 4 × 10^−3^). The frequency of the minor allele “G” of rs7033979 in the intron region of the *ASPN* (NM_001012267.2) gene was lower in patients with KBD than in controls (OR = 0.744, 95% CI = 0.597–0.927, *P* = 8 × 10^−3^). The other 13 OA-related SNPs were not significantly associated with KBD in the population used in this study, but we cannot exclude the possibility that these genes are involved in susceptibility to KBD. Because for most of these genes, only one polymorphism was studied in this study.

**Table 2:** Selected SNPs related to osteoarthritis

Gene	SNP	Related Disease	Reference
A2BP1	rs716508_C/T	Hand osteoarthritis	[34, 35]
ADAM12	rs3740199_C/G	Knee osteoarthritis	[36–38]
	rs1044122_C/T		
	rs1871054_C/T		
ASPN	rs7033979_A/G	Hand, knee, and hip osteoarthritis	[29, 30, 39]
BTNL2	rs10947262_C/T	Knee osteoarthritis	[40]
COG5	rs3757713_G/T	Osteoarthritis	[41, 42]
DUS4L	rs4730250_A/G	Knee osteoarthritis	[43]
FRZB	rs7775-C/G	Hip osteoarthritis in females	[27, 30, 44]
HLA	rs7775228_C/T	Knee osteoarthritis	[12, 40]
IL1B	rs1143634_C/T	Knee and hip osteoarthritis	[45, 46]
	rs1143633_A/G		
RHOB	rs585017_A/G	Knee osteoarthritis	[47, 48]
SMAD3	rs12901499_A/G	Knee, hip, and hand osteoarthritis	[49, 50]
TXNDC3	rs4720262_C/T	Knee osteoarthritis	[47, 51]

**Table 3:** Comparison of allele frequencies of the 15 variants between cases and controls

Gene	SNP	Region/Change	Minor_allele	*P*_allele	MAF Case/Control	*P*_HWE Case/Control	OR (95% CI)
A2BP1	rs716508	intron	C	0.075	0.222/0.304	0.299/0.797	0.651 (0.405–1.046)
ADAM12	rs3740199	p.Gly48Arg	C	0.381	0.431/0.478	0.344/0.582	0.829 (0.545–1.262)
	rs1044122	synonymous	C	0.313	0.392/0.341	0.51/0.502	1.248 (0.811–1.92)
	rs1871054	intron	C	0.807	0.443/0.456	0.579/0.381	0.949 (0.626–1.44)
ASPN	rs7033979	intron	G	8 × 10^−3^	0.157/0.200	0.044/0.048	0.744 (0.597–0.927)
BTNL2	rs10947262	p.Ser188Leu	T	0.925	0.280/0.275	0.691/0.262	1.025 (0.611–1.719)
COG5	rs3757713	intron	G	0.499	0.191/0.22	0.606/0.111	0.838 (0.502–1.399)
DUS4L	rs4730250	intron	G	0.289	0.179/0.227	0.693/0.113	0.746 (0.434–1.284)
FRZB	rs7775	p.Arg324Gly	G	4 × 10^−3^	0.083/0.055	0.330/0.808	1.568 (1.151–2.136)
HLA	rs7775228	-	C	0.321	0.253/0.209	0.861/0.539	1.282 (0.784–2.097)
IL1B	rs1143634	synonymous	T	0.931	0.041/0.039	0.694/0.015	1.048 (0.36–3.054)
	rs1143633	intron	G	0.744	0.483/0.500	0.199/0.292	0.933 (0.614–1.417)
RHOB	rs585017	upstream	G	0.098	0.022/0.055	0.831/0.143	0.382 (0.118–1.242)
SMAD3	rs12901499	intron	G	0.412	0.344/0.386	0.278/0.455	0.836 (0.545–1.282)
TXNDC3	rs4720262	utr-5	T	0.668	0.118/0.133	0.196/0.231	0.875 (0.474–1.614)

*SNP* single-nucleotide polymorphism, *P_allele* the association P value for minor allele, *MAF* Minor Allele Frequency, *P_HWE* the P value of Hardy-Weinberg equilibrium (HWE), *OR* odds ratio for the effect allele, 95% CI 95% confidence interval

### Association of variants in the *COL10A1* and *HABP2* genes with KBD

We carried out Sanger sequencing analyses of *COL10A1* and *HABP2* gene exons with the goal of identifying coding variants that may contribute to KBD risk in the Tibetan population using 849 KBD patients and 565 normal controls. In both KBD patients and controls, genotype distribution did not deviate significantly from the HWE (*P*_HWE > 0.05). In all, 21 and 23 non-synonymous changes were detected in *COL10A1* and *HABP2* genes, respectively (Additional file 3: Table S3). We found three of these variants, rs142463796 (p.Asp128Asn) and rs2228547 (p.Gly545Arg) in the *COL10A1* gene and rs548354451 (p.Asp272Glu) in the *HABP2* gene, were weakly associated with KBD (Table 4). The candidacy of rs142463796 in the *COL10A1* gene is particularly strong given the low *P* value (OR = 9.832, 95% CI = 1.302–74.266, *P* = 6 × 10^−3^), in which the effect is associated with the minor allele “T” (causing aspartic acid to asparagine at amino acid 128 in the *COL10A1* gene). SNP rs2228547 demonstrated a nominally significant association with KBD in the allelic tests (OR = 1.242, 95% CI = 0.999–1.544, *P* = 0.043). The frequency of the minor allele “A” of rs548354451 in the *HABP2* gene differed between KBD patients and controls (OR = 2.813, 95% CI = 1.237–6.397, *P* = 0.010), and this variant led to the replacement of an aspartic acid by a glutamic acid at codon 272 (NP_004123).

**Table 4:** Association between the three SNPs within the *COL10A1* and *HABP2* genes and the presence of KBD revealed in the exon sequencing analysis

Gene	SNP	Amino change	Minor_allele	P_allele	MAF Case/Control	P_HWE Case/Control	OR (95% CI)
COL10A1	rs142463796	p.Asp128Asn	T	6 × 10^−3^	0.011/0.001	0.769/0.981	9.832 (1.302–74.266)
COL10A1	rs2228547	p.Gly545Arg	C	0.043	0.188/0.156	0.460/0.056	1.242 (0.999–1.544)
HABP2	rs548354451	p.Asp272Glu	A	0.010	0.020/0.007	0.566/0.874	2.813 (1.237–6.397)

*SNP* single-nucleotide polymorphism, *P_allele* the association P value for minor allele, *MAF* Minor Allele Frequency, *P_HWE* the P value of Hardy-Weinberg equilibrium (HWE), *OR* odds ratio for the effect allele, 95% CI 95% confidence interval

## Discussion

Although the cause of KBD remains elusive, it may well result from an interaction between genes and environment, which is supported by the following facts. First, moving populations from affected areas to unaffected areas significantly decreased disease prevalence, but KBD could not be abolished completely in the same population [12]. Second, although supplying selenium and/or iodine has produced positive effects in most KBD-affected areas, there are some affected areas where the effects of these supplements have been unclear [23]. Third, not everyone in the same family has KBD, even though they share the same environment, drink and eat the products in the KBD areas, including fungal toxins and are exposed to the same viruses et al [12]. Fourth, cattle in the affected areas do not show any KBD similar phenotypes.

Different genetic variants for each individual might explain why some populations are particularly vulnerable to KBD. KBD and OA have similar clinical manifestations and pathologic changes in the articular cartilage, such as chondrocyte necrosis and apoptosis, matrix degradation and cartilage degeneration [24]. Thus, KBD and OA may share some disease-related genes and pathogenesis. In this study, we investigated whether the 15 SNPs in 12 osteoarthritis-related genes are associated with KBD in a Tibetan population. We found that the SNPs rs7775 in the *FRZB* gene and rs7033979 in the *ASPN* gene showed significant associations with KBD.

The minor allele “G” of rs7775 in the *FRZB* gene was more common in patients with KBD than in controls (OR = 1.568, *P* = 4 × 10^−3^), suggesting that rs7775 might increase susceptibility to KBD. The *FRZB* gene encodes a soluble protein FRZB (also called sFRP-3), which is involved in negative regulation of the WNT signaling pathway. WNT/β-catenin signaling is a powerful stimulator of chondrocyte matrix catabolic action and may be part of mechanisms leading to excessive remodeling and degradation of cartilage matrix in joint pathologies [25]. *Frzb* (−/−) mice have been shown to contribute to cartilage damage by increasing WNT signaling and expression and activity of matrix metalloproteinase 3 (MMP-3) [26]. Several studies have explored the relationship between OA and rs7775 in the *FRZB* gene. By linkage and association studies, Loughlin et al. found that a p.Arg324Gly (rs7775) substitution in the *FRZB* gene was associated with hip OA in females (*n* = 338; *P* = 0.04) [27]. In a Dutch study, the rs7775 (p.Arg324Gly) was found to be associated with generalized osteoarthritis (GOA). Our data suggested for the first time that the rs7775 polymorphism was associated with KBD in the Tibetan population. Asporin (ASPN) is an extracellular matrix (ECM) protein that can bind to TGF-β1 and sequentially inhibit TGF-β/Smad signaling. TGF-β and BMP2 are crucial to differentiation and proliferation of perichondrial cells [28]. ASPN has been found expressed in high proportions in the cartilage of osteoarthritic patients. It acts as a negative regulator of chondrogenesis by inhibiting the action of TGF-β [29]. A meta-analysis conducted by Valdes et al. suggested that an ASPN allele, D13, is protective against the risk of knee OA in Japanese and Caucasians [30]. In this study, the frequency of the minor allele “G” of rs7033979 in the *ASPN* gene was less in KBD patients than in controls (0.157 versus 0.200), suggesting a protective effect of this variant on KBD (OR = 0.744, *P* = 4 × 10^−3^).

In the *COL10A1* and *HABP2* genes’ exon sequencing analysis, we found that two coding variants in the *COL10A1* gene, rs142463796 and rs2228547, were significantly associated with KBD in the Tibetan population studied. The risk allele “T” of rs142463796 (p.Asp128Asn) had a 9.832-fold increased likelihood of developing KBD. *COL10A1* codes for abundant proteins in the extracellular matrix. Abnormal *COL10A1* expression and diverse mutations have been observed in multiple skeletal diseases, such as Cleidocranial Dysplasia (CCD) [31], SMCD [32] and SMD [33], implying a role of *COL10A1* variants in KBD.

**Fig. 2:**
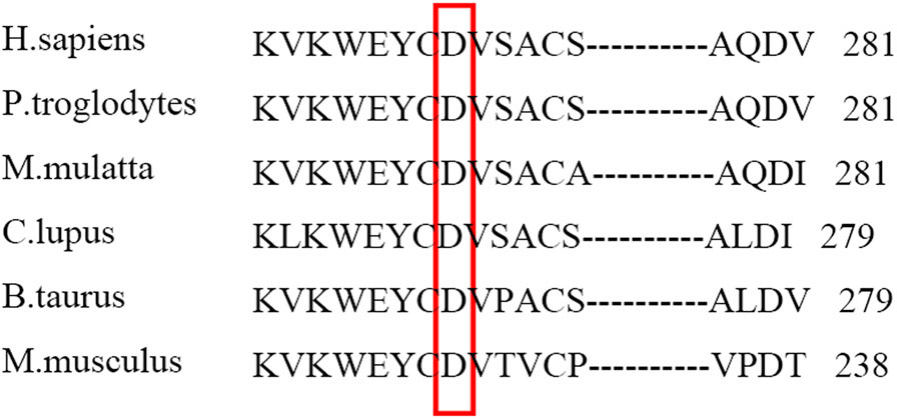
Protein sequence alignment of human HABP2 with its orthologs. The residue of the missense mutation p.Asp272Glu (p.D272E) is highly conserved in Euteleostomi from *H.sapiens* to *M.musculus*

Based on the clinicopathologic role of cartilage cells in KBD patients, we supposed genes involved in extracellular matrix homeostasis in articular cartilage would play an important role in the development of KBD. We explored for the first time the association of variants in the *HABP2* gene with KBD. We noted that the rare variant rs548354451 in the *HABP2* gene was associated with KBD (OR = 2.813, *P* = 0.010). This variant led to the replacement of an aspartic acid by a glutamic acid at codon 272 (p.Asp272Glu), which is involved in a highly evolutionarily conserved residue in Euteleostomi from *H.sapiens* to *M.musculus* (Fig. 2). The protein encoded by the *HABP2* gene is an extracellular serine protease that binds to hyaluronic acid, whose balance is important in maintaining the structure of cartilage and bone formation and, consequently, has been used to treat KBD patients [19].

Overall, we genotyped 15 SNPs in12 candidate genes in Tibetan KBD patients and controls, as well as two genes derived from cartilage-related pathways. Our finding suggested that rs7775 in the *FRZB* gene might increase susceptibility to KBD, while rs7033979 plays a protective role in susceptibility to KBD in the endemic region. Furthermore, genetic variants in the chondrogenesis-related genes *COL10A1* and *HABP2* may play an important role in KBD pathogenesis. The fact that significant differences in genetic variants between cases and controls did not exist after Bonferroni correction (*P* = 0.05/tested loci number) could be explained by the relatively small sample size, with small effects of the variants. Further studies with larger cohorts are needed to confirm the findings of this study.

## Conclusion

In this study, we reported two SNPs of osteoarthritis-related genes, rs7775 in the FRZB gene and rs7033979 in the ASPN gene, showed significant associations with KBD in Tibetans. These findings supporting the possibility of shared genetics etiology between OA and KBD. Moreover, we found that genetic variants in chondrogenesis-related genes COL10A1 and HABP2 may play a role in the risk of developing KBD in the Tibetan population.

## Additional files

Additional file 1: Table S1. Primers of selected SNPs used in this study. (DOC 67 kb)

Additional file 2: Table S2. Primers used for exons sequencing. (DOC 48 kb)

Additional file 3: Table S3. Rare variants detected in the *COL10A1* and *HABP2* exon sequencing of KBD Cases and normal controls. (DOC 80 kb)

## Abbreviations

ASPN: Asporin
CCD: Cleidocranial Dysplasia
COL10A1: Collagen Type X Alpha 1 Chain
FA: Fulvic acid
FRZB: Frizzled-related protein
GPX1: Glutathione peroxidase 1
HABP2: Hyaluronan binding protein 2
HLA-DRB1: Major histocompatibility complex, class II, DR Beta 1
HWE: Hardy-Weinberg equilibrium
KBD: Kashin-Beck disease
OA: Osteoarthritis
RA: Rheumatoid arthritis
SMCD: Schmid-type metaphyseal chondrodysplasia
SMD: Japanese-type spondylometaphyseal dysplasia
SNP: Single-nucleotide polymorphisms
