# Supplementary material for: Association study of candidate genes for susceptibility to Kashin-Beck disease in a Tibetan population
Source: BMC Med Genet. 2017 Jun 26;18:69. doi: 10.1186/s12881-017-0423-6 (PMC5485673; doi:10.1186/s12881-017-0423-6)
Supplement: Supplementary file 1 — Primers of selected SNPs used in this study. (DOC 67 kb) [file 12881_2017_423_MOESM1_ESM.doc]

**Table S1.** Primers of selected SNPs used in this study.

| **Gene** | **SNP** | **Primers** |
| --- | --- | --- |
| A2BP1 | rs716508-F | CATTGCTCTAAAACAGGATACTGC |
|  | rs716508-R | ATACCCTTGGGTGAGGTGTG |
|  | rs716508-S60-C/T | tctccctgcactagaatgcaacatctgtaagaacaaggattacatttttcccatacatgg |
|  |  |  |
| ADAM12 | rs3740199-F | AAAAGCTTCGGCAGTCTCAA |
|  | rs3740199-R | CTTTTGATTCAGGGGTGAGC |
|  | rs3740199-S60-C/G | aaggagaggaactcaccttggagtcgaagctcttcactgggatccagaggtccccactcc |
|  |  |  |
|  | rs1044122-F | ACATCAGCAGACCCCTCAAC |
|  | rs1044122-R | AGGCACCGAGAAGTTAAGCA |
|  | rs1044122-S20-C/T | cttcctcccctccaccgggc |
|  |  |  |
|  | rs1871054-F | AAAACACTTGCTGGGACAGG |
|  | rs1871054-R | AAGGAGGCTGCATCATGAAC |
|  | rs1871054-S50-C/T | gtagcattgccatctggctgcccatggctctccagagtagcacaggccac |
|  |  |  |
| ASPN | rs7033979-F | TTTTGTGGATTTGGCTGACA |
|  | rs7033979-R | TCAAGTTTTGCTGCCTTTTC |
|  | rs7033979-S60-A/G | cagcctttcacctgagggtcttagcattcatgcacgattgttgcctagatccattctgtc |
|  |  |  |
| BTNL2 | rs10947262-F | GGTGTCCAGCCTGTCAAAAT |
|  | rs10947262-R | CATGAGAATAAATGACTCAAGGAA |
|  | rs10947262-S50-C/T | ttcaaaaatgtgggtgaggttgctgtctgtcacctaccagctatgtgatt |
|  |  |  |
|  |  |  |
| COG5 | rs3757713-F | TCTGGAGGTTCAAGGTGGAG |
|  | rs3757713-R | ACCAGGACCCTAGGCTTGAC |
|  | rs3757713-S20-G/T | agcttggtacctggctataa |
|  |  |  |
| DUS4L | rs4730250-F | AGAAATGTTTCATTCTGGACAGC |
|  | rs4730250-R | AGGAGTTACAACCACCACCA |
|  | rs4730250-S50-A/G | aatgattaatgaactaagatgaaatttcccttttatgctctgacttttta |
|  |  |  |
| FRZB | rs7775-F | CTTGCAGTCTGACCCAGTTG |
|  | rs7775-R | GCAGAAACCAAAAGAGAAACAGA |
|  | rs7775-S40-C/G | gagtcagaagtctggcaggaactcgaacccccggcaagca |
|  |  |  |
| HLA | rs7775228-F | TCCAAGATCCAGTCAGTAGTGAAG |
|  | rs7775228-R | GCTTGGACTGCCAGAGACA |
|  | rs7775228-S50-C/T | ttgcaggaggaaaggaactatctgggtatggaaaaggttactaggcaaga |
|  |  |  |
| IL1B | rs1143634-F | TGTTCTTAGCCACCCCACTC |
|  | rs1143634-R | CGTGCACATAAGCCTCGTTA |
|  | rs1143634-S20-C/T | atttcagaacctatcttctt |
|  |  |  |
|  | rs1143633-F | TGTTCTTAGCCACCCCACTC |
|  | rs1143633-R | TCGTTATCCCATGTGTCGAA |
|  | rs1143633-S40-A/G | cgtatatgctcaggtgtcctccaagaaatcaaattttgcc |
|  |  |  |
| TXNDC3 | rs4720262-F | CCCTCTTCTTCCTTTTGCTG |
|  | rs4720262-R | CTGGGGCACGACATAAACTT |
|  | rs4720262-S40-C/T | ttagatcctctgggcctgttccttccttttctttaaacgt |
|  |  |  |
| RHOB | rs585017-F | GCTCCAGACAGCCAGCTC |
|  | rs585017-R | TATAGCCGTCCAATGGGAAA |
|  | rs585017-S50-A/G | aaggaggggacccgggtaccgccagagccccgcagcggcagcagcagcgc |
|  |  |  |
| SMAD3 | rs12901499-F | CCGGAGTTATCCAGGTCAGA |
|  | rs12901499-R | CCCCACTTAGTTTCCCAAGG |
|  | rs12901499-S40-A/G | ccatgtggacagctttaggctatgtctggagcaggttgaa |
