# Supplementary material for: Association study of candidate genes for susceptibility to Kashin-Beck disease in a Tibetan population
Source: BMC Med Genet. 2017 Jun 26;18:69. doi: 10.1186/s12881-017-0423-6 (PMC5485673; doi:10.1186/s12881-017-0423-6)
Supplement: Supplementary file 2 — Primers used for exons sequencing. (DOC 48 kb) [file 12881_2017_423_MOESM2_ESM.doc]

**Table S2. Primers used for exons sequencing.**

| **Gene** | **Exon** | **Left_Primer** | **Right_Primer** | **Size** |
| --- | --- | --- | --- | --- |
| HABP2 | 1 | ccagccccaaagttgcc | ctcatcactcccagcctagc | 253 |
|  | 2 | ctggctgacagctaaggagc | cttttcaccagcccagaacc | 176 |
|  | 3 | aaataaaatgctggcctatttg | gtcccacatacactccgctc | 248 |
|  | 4 | ggggcgcttctcacctg | cgcagtgctttctcacacag | 243 |
|  | 5 | tgtatccctctaaggaaaatgtg | tgcaggtacaagatagggcac | 233 |
|  | 6 | gccaccaatgtctccttgtc | agaggaaagggttccagagg | 260 |
|  | 7 | gcaggtccagttctctcacc | ggaggctgatgaatttcaatg | 351 |
|  | 8 | tccaaaggttctttaataagatccag | ccctacaaatctccatcccc | 228 |
|  | 9 | ctgaggaactggagggagg | atgctgctcagaggggaag | 387 |
|  | 10 | ggctgagtctgcagaaggtc | ccaaacttcagctttctggtc | 308 |
|  | 11 | caagttggagctggtgcttc | gtttggcctttcttctctgg | 272 |
|  | 12 | gttggtgccaccctggtc | ctgcctagtgaaacatcctgc | 263 |
|  | 13 | ccctggagagaggtggg | CTCCCGGTGTCAGGGTG | 294 |
|  | 13-2 | AAGCAGAGACAACTGCCACC | GAGGCCAGAGAGGAAGTTTG | 460 |
|  | 13-3 | AAAGGAAGATCTGGGATGGG | TGCATTTTGAACCCTACTTTG | 468 |
|  | 13-4 | CCTTTTCCCCTCTTCTACCC | gtagcaagctgcaaagccac | 462 |
|  | 1 | accaactttgaggggaggag | ttttggagtgcattttagtagaaac | 312 |
| COL10A1 | 2 | cagacatccaatcctaatttctg | gtgcaaaagcagcatctgag | 717 |
|  | 3_1 | ttctctttaatgtcactaaccatcc | ACGACCAGGAGCACCATATC | 501 |
|  | 3_2 | TGGACAACAGGGACCCAC | CCCTTTGGCACCTGGAC | 496 |
|  | 3_3 | CTCCAGGAATAGCTGGGC | CTGGTATTCCAGGGGCAC | 498 |
|  | 3_4 | TGATCCTGGAGTTGGAGGAC | TTTTATCAAATGGTATGGGAGTTC | 491 |
|  | 3_5 | CCCTCTTGTTAGTGCCAACC | GCATTTTGTAGGGTGGGGTAG | 505 |
|  | 3_6 | CCTGAAAAGTGAGCAGCAAC | CATGTGCTAATGTTCTGTAAATCC | 691 |
|  | 3_7 | TGCTTTCATCAATGAACCTTTTC | tgggaataaagatgcatcacc | 563 |
