# Supplementary material for: Association study of candidate genes for susceptibility to Kashin-Beck disease in a Tibetan population
Source: BMC Med Genet. 2017 Jun 26;18:69. doi: 10.1186/s12881-017-0423-6 (PMC5485673; doi:10.1186/s12881-017-0423-6)
Supplement: Supplementary file 3 — Rare variants detected in the COL10A1 and HABP2 exon sequencing of KBD Cases and normal controls. (DOC 80 kb) [file 12881_2017_423_MOESM3_ESM.doc]

**Table S3**. Rare variants detected in the *COL10A1* and *HABP2* exon sequencing of KBD Cases and normal controls.

| **Gene** | **Location** | **Region** | **Base_change** | **Amino_change** | **SNP** | **MAF_Case** | **MAF_Control** | **P_allele** | **OR（95% CI）** |
| --- | --- | --- | --- | --- | --- | --- | --- | --- | --- |
| COL10A1 | chr06:116441239 | exon3 | c.G2040C | p.M680I | rs200235459 | 0.0006 | 0.0000 | 0.4145 | - |
| COL10A1 | chr07:116441303 | exon3 | c.C1976T | p.S659L | . | 0.0000 | 0.0009 | 0.2202 | - |
| COL10A1 | chr08:116441597 | exon3 | c.C1682T | p.A561V | rs201416912 | 0.0012 | 0.0018 | 0.6816 | 0.6651(0.0935-4.7286) |
| **COL10A1** | **chr06:116441646** | **exon3** | **c.G1633C** | **p.G545R** | **rs2228547** | **0.1880** | **0.1561** | **0.0432** | **1.2511(1.0066-1.555)** |
| COL10A1 | chr06:116442041 | exon3 | c.G1238T | p.G413V | . | 0.0006 | 0.0009 | 0.7718 | 0.6653(0.0416-10.6478) |
| COL10A1 | chr06:116442098 | exon3 | c.C1181G | p.P394R | rs201458750 | 0.0053 | 0.0053 | 0.9973 | 0.9982(0.3543-2.8122) |
| COL10A1 | chr06:116442132 | exon3 | c.G1147A | p.G383R | rs145378345 | 0.0035 | 0.0035 | 0.9978 | 0.9982(0.2811-3.5454) |
| COL10A1 | chr06:116442414 | exon3 | c.A865G | p.I289V | rs558704595 | 0.0018 | 0.0018 | 0.9985 | 0.9982(0.1665-5.9837) |
| COL10A1 | chr06:116442491 | exon3 | c.T788C | p.I263T | rs200461789 | 0.0006 | 0.0000 | 0.4145 | - |
| COL10A1 | chr06:116442533 | exon3 | c.C746G | p.P249R | rs188598435 | 0.0012 | 0.0009 | 0.8147 | 1.3314(0.1206-14.7006) |
| COL10A1 | chr06:116442588 | exon3 | c.C691T | p.P231S |  | 0.0000 | 0.0009 | 0.2202 | - |
| COL10A1 | chr06:116442614 | exon3 | c.G665A | p.R222K | rs772549860 | 0.0012 | 0.0009 | 0.8147 | 1.3314(0.1206-14.7006) |
| COL10A1 | chr06:116442686 | exon3 | c.G593A | p.R198H | rs148785195 | 0.0006 | 0.0009 | 0.7718 | 0.6653(0.0416-10.6478) |
| COL10A1 | chr06:116442687 | exon3 | c.C592T | p.R198C | rs769103179 | 0.0012 | 0.0009 | 0.8147 | 1.3314(0.1206-14.7006) |
| COL10A1 | chr06:116442867 | exon3 | c.C412T | p.R138W | rs769103179 | 0.0000 | 0.0009 | 0.2202 | - |
| **COL10A1** | **chr06:116442897** | **exon3** | **c.G382A** | **p.D128N** | **rs142463796** | **0.0105** | **0.0011** | **0.0064** | **9.8322(1.3017-74.2657)** |
| COL10A1 | chr06:116442980 | exon3 | c.C299T | p.P100L | rs538524572 | 0.0006 | 0.0000 | 0.4145 | - |
| COL10A1 | chr06:116446549 | exon2 | c.C107T | p.P36L | rs778850616 | 0.0000 | 0.0009 | 0.2202 | - |
| COL10A1 | chr06:116446576 | exon2 | c.T80C | p.M27T | rs1064583 | 0.2479 | 0.2487 | 0.9647 | 0.9961(0.8369-1.1856) |
| COL10A1 | chr06:116446601 | exon2 | c.G55A | p.V19M | . | 0.0000 | 0.0009 | 0.2202 | - |
| COL10A1 | chr06:116446654 | exon2 | c.T2C | p.M1T | rs748339808 | 0.0006 | 0.0000 | 0.4145 | - |
| HABP2 | chr10:115312899 | exon1 | c.G19C | p.D7H | . | 0.0006 | 0.0000 | 0.4145 | - |
| HABP2 | chr10:115312926 | exon1 | c.C46G | p.L16V | rs567136227 | 0.0012 | 0.0009 | 0.8147 | 1.3314(0.1206-14.7006) |
| HABP2 | chr10:115334110 | exon3 | c.A169G | p.S57G | rs752239121 | 0.0006 | 0.0009 | 0.7718 | 0.6653(0.0416-10.6478) |
| HABP2 | chr10:115334144 | exon3 | c.A203G | p.Y68C | rs200071983 | 0.0006 | 0.0009 | 0.7718 | 0.6653(0.0416-10.6478) |
| HABP2 | chr10:115334164 | exon3 | c.G223T | p.D75Y | rs542838125 | 0.0006 | 0.0000 | 0.4145 | - |
| HABP2 | chr10:115337823 | exon6 | c.G487A | p.A163T | rs374554126 | 0.0000 | 0.0009 | 0.2202 | - |
| HABP2 | chr10:115337835 | exon6 | c.C499T | p.R167W | rs145980446 | 0.0000 | 0.0009 | 0.2202 | - |
| HABP2 | chr10:115338398 | exon7 | c.G581A | p.C194Y | rs545209433 | 0.0006 | 0.0000 | 0.4145 | - |
| HABP2 | chr10:115338449 | exon7 | c.A632C | p.N211T | rs749339659 | 0.0006 | 0.0009 | 0.7718 | 0.6653(0.0416-10.6478) |
| HABP2 | chr10:115338476 | exon7 | c.C659G | p.S220C | rs201294830 | 0.0006 | 0.0000 | 0.4145 | - |
| HABP2 | chr10:115338482 | exon7 | c.T665A | p.L222H | rs533439977 | 0.0006 | 0.0009 | 0.7718 | 0.6653(0.0416-10.6478) |
| HABP2 | chr10:115340416 | exon8 | c.G803A | p.W268X | rs200879638 | 0.0000 | 0.0009 | 0.2202 | - |
| **HABP2** | **chr10:115340429** | **exon8** | **c.T816A** | **p.D272E** | **rs548354451** | **0.0198** | **0.0071** | **0.0100** | **2.8125(1.2366-6.3967)** |
| HABP2 | chr10:115341722 | exon9 | c.G926A | p.R309K | . | 0.0000 | 0.0009 | 0.2202 | - |
| HABP2 | chr10:115341743 | exon9 | c.G947A | p.G316E | rs138864377 | 0.0041 | 0.0035 | 0.8074 | 1.1653(0.3403-3.9899) |
| HABP2 | chr10:115341788 | exon9 | c.T992C | p.L331P | rs754664748 | 0.0006 | 0.0000 | 0.4145 | - |
| HABP2 | chr10:115341802 | exon9 | c.C1006T | p.P336S | rs570843767 | 0.0006 | 0.0009 | 0.7718 | 0.6653(0.0416-10.6478) |
| HABP2 | chr10:115343057 | exon10 | c.G1177C | p.E393Q | rs11575688 | 0.0000 | 0.0009 | 0.2202 | - |
| HABP2 | chr10:115343085 | exon10 | c.A1205G | p.N402S | rs745708955 | 0.0012 | 0.0000 | 0.2485 | - |
| HABP2 | chr10:115344036 | exon11 | c.A1367G | p.E456G | rs554917785 | 0.0006 | 0.0018 | 0.3447 | 0.3324(0.0301-3.6697) |
| HABP2 | chr10:115345623 | exon12 | c.C1444T | p.L482F | . | 0.0012 | 0.0035 | 0.1812 | 0.332(0.0607-1.8154) |
| HABP2 | chr10:115345651 | exon12 | c.T1472C | p.M491T | . | 0.0006 | 0.0000 | 0.4145 | - |
| HABP2 | chr10:115348046 | exon13 | c.G1601A | p.G534E | rs7080536 | 0.0000 | 0.0009 | 0.2202 | - |

*SNP:* single-nucleotide polymorphism, *P_allele:* the association P value for minor allele, *MAF*: Minor Allele Frequency, *P_HWE*: the P value of Hardy-Weinberg equilibrium (HWE), *OR*: odds ratio for the effect allele, 95% CI: 95% confidence interval.
